# Real-World Safety of an Implantable Continuous Glucose Sensor Over Multiple Cycles of Use: A Post-Market Registry Study

**DOI:** 10.1089/dia.2019.0159

**Published:** 2020-01-02

**Authors:** Dorothee Deiss, Concetta Irace, Grace Carlson, Katherine S. Tweden, Francine R. Kaufman

**Affiliations:** ^1^ Center for Endocrinology and Diabetology, Medicover-Berlin Mitte, Berlin, Germany.; ^2^ Department of Health Science, University Magna Græcia, Catanzaro, Italy.; ^3^ Carlson Consulting, San Francisco, California.; ^4^ Senseonics, Incorporated, Germantown, Maryland.

**Keywords:** Continuous glucose monitoring, Sensor, Implantable, Type 1 diabetes, Type 2 diabetes, Safety

## Abstract

Previously, the safety and accuracy of the Eversense continuous glucose monitoring (CGM) system were characterized in three pivotal trials among individuals with type 1 diabetes (T1D) and type 2 diabetes (T2D) with a single 90- or 180-day sensor insertion–removal cycle. The Post-Market Clinical Follow-up (PMCF) registry is a prospective study evaluating the long-term safety and performance of the Eversense CGM system over multiple sensor insertion–removal cycles among adults with T1D and T2D. All patients who had a sensor subcutaneously implanted across 534 participating centers in Europe and South Africa from June 2016 to August 2018 were enrolled. Adverse events (AEs) were recorded at each visit and patients were instructed to inform their clinic if they experienced any AEs between visits. AEs were adjudicated for relatedness to the device, procedure, or drug (dexamethasone acetate). The primary safety endpoint was the rate of related serious adverse events (SAEs) through four sensor insertion–removal cycles. The registry enrolled 3023 patients. As of last follow-up, 5417 sensors had been inserted with a total of 1260 patient-years (PYs) of follow-up: 969 patients had used the system for at least 6 months and 173 patients had used the system for at least 1 year. No related SAEs were reported. The most frequently reported related AEs were sensor location site infection (0.96%; 2.46 events per 100 PYs), inability to remove the sensor upon first attempt (0.76%; 1.90 events per 100 PYs), and adhesive patch location site irritation (0.66%; 1.59 events per 100 PYs). One nonserious allergic reaction to lidocaine was reported, which resolved with administration of an antihistamine. The full intended sensor life was achieved by 91% of 90-day sensors and 75% of 180-day sensors. The PMCF registry provides real-world evidence that the Eversense CGM system is safe over multiple cycles of use.

## Introduction

Continuous glucose monitoring (CGM) systems improve glycemic control and reduce the incidence and duration of hypoglycemia among patients with type 1 diabetes (T1D) and type 2 diabetes (T2D).^1–4^ Although the uptake of traditional transcutaneous CGM systems has substantially increased in recent years, alternative solutions are needed to address the reasons why patients either do not start CGM or discontinue after the first year.^5–7^

The fully implantable fluorescence-based Eversense CGM system was designed to address several of the limitations of traditional CGM systems. Initial approval of the Eversense system was granted in Europe in May 2016 with an up to 90-day sensor wear time (Eversense^®^ CGM System), which was extended to an up to 180-day sensor wear time (Eversense^®^ XL CGM System) in September 2017. The 90-day system was approved for use in the United States in June 2018. Regulatory approval in Europe and the United States was based on three pivotal clinical studies, which evaluated the accuracy and safety of the system over a single insertion–removal cycle up to 180 days (PRECISE) or up to 90 days (PRECISE II and PRECISION). These studies enrolled 206 patients who had 335 sensor insertion–removal cycles and were followed for a cumulative total of 61 patient-years (PYs).^8–10^

The safety profile of the Eversense CGM system over a single insertion–removal cycle was favorable in all three pivotal studies and compared favorably with traditional transcutaneous CGM systems.^8–10^ Device- or procedure-related adverse events (AEs) were relatively infrequent (12.6% of patients). AEs were generally mild (78% of events) and transient in nature (66% of events resolved in <2 weeks), and typically resolved with either no or minimal intervention (83% of events resolved with either no intervention, over-the-counter treatments, or laboratory assessments). The most common device- or procedure-related AEs in a pooled analysis of pivotal studies were pain/discomfort (2.9% of patients), redness/erythema at the sensor insertion site (2.4% of patients), incision site infection (1.5% of patients), and inability to remove the sensor on the first attempt (1.5% of patients).^11^ The primary limitation of the safety database from the pivotal studies is that only a single 90- or 180-day insertion–removal cycle was evaluated.

The goal of this registry study was to evaluate the long-term safety of the Eversense CGM system in a large real-world patient population following multiple insertion–removal cycles.

## Methods

### Study device

The Eversense CGM system has been previously described in detail.^8,9^ The system comprises three components: a small fully implantable fluorescence-based glucose sensor, a removable smart transmitter, and a mobile application that allows for real-time monitoring of current and historical glucose values on a mobile device. The 90- and 180-day sensors have similar components, including a silicone rubber dexamethasone acetate-eluting ring that contains 1.75 mg of dexamethasone acetate to reduce the foreign body response to the device. Sensors are placed in the subcutaneous tissue of the upper arm under local anesthesia with lidocaine. The placement site is closed using Steri-Strips™ without the need for suturing. The patient-contacting material in the adhesive for the removable smart transmitter is silicone.

The transmitter wirelessly communicates through NFC with the sensor and sends data to the mobile application through low energy Bluetooth. The CGM system provides hypoglycemia and hyperglycemia threshold, rate-of-change, and predictive alerts. Two sensor calibrations per day with blood glucose meter readings are required. The sensor operating life is up to either 90 or 180 days, depending on the sensor, or until the fluorescence intensity data indicate that the sensor sensitivity to glucose is inadequate to maintain high accuracy.

### Study design and participants

The Post-Market Clinical Follow-up (PMCF) registry was a prospective registry study among adult participants aged 18 years or older with T1D and T2D across 534 centers in Europe and South Africa. All patients who received the Eversense CGM system at the participating centers were to be enrolled until at least 100 patients had reached four sensor insertion–removal cycles. Consistent with the device label, patients were not candidates for the system if they required a planned MRI during the period of sensor wear, were critically ill or hospitalized, had a known contradiction to dexamethasone, required intravenous mannitol or mannitol irrigation solutions, or were pregnant. The PMCF registry was conducted in compliance with the Declaration of Helsinki, according to the European medical device regulations (MEDDEV 2.12–2), and applicable local requirements for prospective registry data collection. Owing to the registry nature of the study, identifying patient information (e.g., age, gender, T1D/T2D status) was removed before being transferred to the study database.

### Procedures

Before the first insertion, patients were trained on the use of the device. Follow-up visits were scheduled every 90 or 180 days, depending on the sensor, for removal of the expired sensor and insertion of a new sensor in the opposite arm.

Investigators documented all AEs that were thought to be potentially related to the device, procedure, or drug (dexamethasone acetate) occurring in the clinic and during home use. All current and previous sensor insertion–removal sites and the surrounding area were assessed by the health care provider at each placement and visit. Patients were asked to provide information on any AEs, health-related problems, and changes in health status at each clinic visit. Patients were also instructed to contact the clinic regarding AEs that occurred between visits.

### Outcome measures and statistical methods

An AE was defined as any untoward medical occurrence, unintended disease or injury, or untoward clinical signs (including abnormal laboratory findings) in patients, users, or other persons, whether or not related to the medical device. A serious adverse event (SAE) was defined as an AE that led to death, led to serious deterioration in the health of the patient requiring medical assistance including emergency medical services and/or hospitalization, or led to fetal distress, fetal death, or a congenital abnormality or birth defect. AEs were adjudicated for relatedness to the device, procedure, or drug by a medical monitor.

The primary safety endpoint of the study was the rate of related SAEs through four insertion–removal cycles. The primary safety endpoint was to be evaluated against an 8% performance goal using the Kaplan–Meier method. Power analysis determined that a sample size of 100 patients through four insertion–removal cycles would provide ∼80% power to evaluate the primary safety endpoint.

Sensor survival through the intended sensor life was evaluated using the Kaplan–Meier method for both the 90- and 180-day sensors.

## Results

### Study participants and duration of exposure

The PMCF registry enrolled 3023 patients across 534 participating centers in 14 European countries and South Africa from June 2016 to August 2018. As of August 2018, 5417 sensors had been inserted with a cumulative follow-up of 1260 PYs: 1320 patients (44%) had >1 sensor insertion–removal cycle and 280 patients (9%) had reached their fourth insertion–removal cycle. By last follow-up, 337 patients (11%) had discontinued use of the system. The most common reasons for discontinuation were unknown (*n* = 108, 32%), lack of medical reimbursement (*n* = 97, 29%), and temporary discontinuation for prescription order or availability of the CGM system in their country (*n* = 65, 19%).

### Safety results

One hundred thirty-three AEs were reported of which 117 were adjudicated as related (*n* = 85 procedure, *n* = 22 device, *n* = 6 drug, and *n* = 4 device/procedure). Table 1 provides a summary of related AEs overall and by sensor insertion–removal cycle (Note: The 16 AEs that were adjudicated as not related are listed in the footer of Table 1).

**Table 1.** Summary of Related Adverse Events by Sensor Insertion–Removal Cycle and Incidence Rates per 100 Patient-Years

*Related AE*	*n* (%) by sensor insertion–removal cycle								Overall		
	1 (*N* = 3023)	2 (*N* = 1320)	3 (*N* = 634)	4 (*N* = 280)	5 (*N* = 94)	6 (*N* = 44)	7 (*N* = 19)	8 (*N* = 3)	Events	*n* (%) of subjects	Rate per 100 PY
Sensor location site infection	18 (0.60)	7 (0.53)	5 (0.79)	0	0	1 (2.27)	0	0	31	29 (0.96)	2.46
Unable to remove sensor at first attempt	17 (0.56)	5 (0.38)	0	2 (0.71)	0	0	0	0	24	23 (0.76)	1.90
Adhesive patch location site irritation	14 (0.46)	2 (0.15)	3 (0.47)	1 (0.36)	0	0	0	0	20	20 (0.66)	1.59
Prolonged wound healing after procedure	4 (0.13)	1 (0.08)	1 (0.16)	0	0	0	0	0	6	6 (0.20)	0.48
Sensor location site redness/reaction to dressing	5 (0.17)	1 (0.08)	0	0	0	0	0	0	6	6 (0.20)	0.48
Sensor location site pain/discomfort	3 (0.10)	2 (0.15)	0	0	0	0	0	0	5	5 (0.17)	0.40
Bruising	3 (0.10)	1 (0.08)	0	1 (0.36)	0	0	0	0	5	4 (0.13)	0.40
Sensor broke during removal	4 (0.13)	0	0	0	0	0	0	0	4	4 (0.13)	0.32
Skin atrophy and discoloration	3 (0.10)	1 (0.08)	0	0	0	0	0	0	4	4 (0.13)	0.32
Hematoma	1 (0.03)	1 (0.08)	0	1 (0.36)	0	0	0	0	3	3 (0.10)	0.24
Sensor location irritation/inflammation	1 (0.03)	1 (0.08)	1 (0.16)	0	0	0	0	0	3	3 (0.10)	0.24
Skin discoloration	1 (0.03)	1 (0.08)	0	0	0	0	0	0	2	2 (0.07)	0.16
Skin atrophy over sensor	1 (0.03)	0	0	0	0	0	0	0	1	1 (0.03)	0.08
Patient fainted during procedure	1 (0.03)	0	0	0	0	0	0	0	1	1 (0.03)	0.08
Allergy to lidocaine	0	0	1 (0.16)	0	0	0	0	0	1	1 (0.03)	0.08
Wound dehiscence	1 (0.03)	0	0	0	0	0	0	0	1	1 (0.03)	0.08

The following 16 AEs were adjudicated as not related: hypoglycemia (*n* = 6), hyperglycemia (*n* = 3), arm weakness (*n* = 1), edema (*n* = 1), myocardial infarction (*n* = 1), negative feelings (*n* = 1), rash (*n* = 1), skin irritation (*n* = 1), and tonsillitis (*n* = 1).

AEs, adverse events; PY, patient-years.

No related SAEs were reported through four insertion–removal cycles (0%; 95% confidence interval [CI] 0%–4.4%), which met the performance goal for the primary safety endpoint (Note that the 95% CI was calculated using the exact binomial method since a CI by the Kaplan–Meier method could not be calculated without any events.). No clinically significant differences in the incidence rates of related AEs were observed over multiple sensor insertion–removal cycles.

The most commonly reported related AE was infection at the sensor site. Thirty-one cases of infection were reported (*n* = 29 patients [0.96%]; 2.46 events per 100 PYs). Twenty cases resolved with sensor removal with or without antibiotics, seven cases resolved with local/antibiotic treatment without sensor removal, two cases resolved without information on intervention, and information on resolution was not available in two cases. None of the cases of sensor site infection required hospitalization or administration of systemic antibiotics.

The first attempt of sensor removal was not successful in 24 cases (*n* = 23 patients [0.76%]; 1.90 events per 100 PYs). Twenty sensors were successfully removed during the second attempt by the attending physician or a surgeon; no general anesthesia was required for these secondary procedures. Information on the secondary removal procedure was not available in four cases despite multiple attempts by study personnel to contact the clinical sites. Investigators did not report any other AEs resulting from the unsuccessful first attempts at sensor removal.

Adhesive patch irritation was reported in 20 cases (*n* = 20 patients [0.66%]; 1.59 events per 100 PYs). Fifteen events were reported as resolved and patients continued to use the CGM system, one event resolved with explant for concomitant infection, and information on resolution was not available in four cases.

All other related AEs occurred in six or fewer cases at a rate of 0.20% (0.48 events per 100 PYs) or less.

### Sensor longevity

The Kaplan–Meier rate for sensor survival through intended sensor life was 91% for the 90-day sensor and 75% for the 180-day sensor.

## Discussion

The results of this large registry study demonstrated a favorable safety profile for the Eversense CGM system under real-world use in 3023 patients for 5417 sensor insertion–removal cycles, and for a total of 1260 PYs of device exposure. No related SAEs were reported. The incidence of related AEs over multiple insertion–removal cycles was low. The overall safety profile of the Eversense CGM system in this registry was consistent with the three pivotal trials, which evaluated safety over a single insertion–removal cycle.^8–10^

The most commonly reported related AE, infection at the sensor site, was reported at a rate of 0.96% or 2.46 events per 100 PYs. The rate of infection observed in the registry is considerably lower than the rate of 7.3–11.5 per 100 PYs reported for insulin infusion sets.^12^ In all cases wherein information was available, complete resolution was achieved with a course of antibiotics or with sensor removal.

Inability to remove the sensor on the first attempt occurred in 24 cases. Although such events inconvenience the patient and health care provider, none of the events were serious and the sensor was successfully removed on the second attempt in all cases wherein information was available.

Adhesive patch irritation occurred in 20 cases or 0.66% of all patients. In all cases wherein information was available, the events resolved and the patients continued to use the system, suggesting that the benefits of the device outweighed the inconvenience of transient adhesive irritation. The transient skin reactions observed with Eversense are notably less clinically significant than the allergic contact dermatitis skin reactions that have been reported for transcutaneous CGM systems that contain isobornyl acrylate.^13^

A particular strength of this registry was the evaluation of a large number of patients under real-world use conditions. Given the self-reported nature of data collection in the registry, it is possible that some AEs that occurred between visits were not captured. However, it is unlikely that a serious event related to the device occurred without the clinic's knowledge. Another limitation of the study was the inability to collect information on resolution of some of the AEs despite multiple attempts by study personnel to contact the clinical sites.

Overall, the PMCF registry demonstrated that the safety profile of the Eversense CGM system is consistent over multiple cycles of sensor insertion–removal in patients with T1D and T2D.
